# Signal Distortion: How Intracellular Pathogens Alter Host Cell Fate by Modulating NF-κB Dynamics

**DOI:** 10.3389/fimmu.2018.02962

**Published:** 2018-12-14

**Authors:** Rachel H. Nelson, David E. Nelson

**Affiliations:** ^1^Cellular Generation and Phenotyping Core Facility, Wellcome Sanger Institute, Cambridge, United Kingdom; ^2^Department of Biology, Middle Tennessee State University, Murfreesboro, TN, United States

**Keywords:** NF-κB, dynamics, live cell imaging, macrophage, host:pathogen interactions, innate immunity

## Abstract

By uncovering complex dynamics in the expression or localization of transcriptional regulators in single cells that were otherwise hidden at the population level, live cell imaging has transformed our understanding of how cells sense and orchestrate appropriate responses to changes in their internal state or extracellular environment. This has proved particularly true for the nuclear factor-kappaB (NF-κB) family of transcription factors, key regulators of the inflammatory response and innate immune function, which are capable of encoding information about the mode and intensity of stimuli in the dynamics of NF-κB nuclear accumulation and loss. While live cell imaging continues to serve as a useful tool in ongoing efforts to characterize the feedbacks that shape these dynamics and to connect dynamics to downstream gene expression, it is also proving invaluable for recent studies that seek to determine how intracellular pathogens subvert NF-κB signaling to survive and replicate within host cells by providing quantitative information about the pathogen and changes in NF-κB activity during different stages of an infection. Here, we provide a brief overview of NF-κB signaling in innate immune cells and review recent literature that uses live imaging to investigate the mechanisms by which bacterial and yeast pathogens modulate NF-κB in a variety of different host cell types to evade destruction or maintain the viability of an intracellular growth niche.

## Introduction

The nuclear factor-kappa B (NF-κB) pathway is considered a master regulator of inflammation and is intimately involved in the cellular response to infection (1). Similar to other mammalian transcription factor pathways, such as p53 (2–4), and NFAT (5), the NF-κB pathway can exhibit distinct dynamic responses to different stimuli (6–9). These dynamics, which include damped oscillations (7), allow cells to encode complex information about the modality, concentration, and duration of a particular stimulus in the amplitude, frequency, and persistence of oscillations (6, 10, 11). These dynamics are essentially decoded at the level of gene expression with different patterns of behavior leading to differing cell fates and phenotypes (10, 12, 13). This phenomenon, which is often referred to as dynamic multiplexing, allows cells to efficiently use a limited number of signaling pathways to deal with highly complex signaling environments (11). The dynamic behavior of the NF-κB pathway can be challenging to study using standard biochemical techniques that use population averaging because the responses of individual cells to a given stimulus can differ markedly (7, 9). This may be due to the difficult to control effects of paracrine and autocrine signaling (12), inherent differences between cells and the signaling history of the cell (extrinsic noise), and the stochasticity of certain elements of the signaling pathway (e.g., transcription and translation; intrinsic noise) (8, 14, 15). For these reasons, live cell imaging (often in combination with mathematical modeling) has become an invaluable tool for studying NF-κB signaling (16, 17), and has been used to characterize the specific feedbacks that shape the behavior of the pathway (6, 7, 18, 19). For similar reasons, live cell imaging is being increasingly used to improve our understanding of the role NF-κB signaling plays during infection with intracellular pathogens (20–23). In tissue culture models of infection, only a fraction of the cells within the population may become infected and this will occur at different times between cells making it difficult to build an accurate picture of how NF-κB signaling is affected during each stage of the pathogenic process. Live cell imaging provides a means to deconvolve events occurring during different stages of an infection (20), distinguish between non-infected and infected cells (23), as well as keeping track of changes in intracellular microbial burden within individual cells (22).

In the following review, we will provide a brief overview of NF-κB signaling and describe how live cell microscopy has been used to investigate the capacity of the pathway to encode information about the signaling environment of the cell in the dynamics of NF-κB transcription factors. We will discuss the duality of NF-κB signaling within the context of host:pathogen interactions and how it can both aid and hinder the response to an infection. Finally, we describe how recent live cell studies have provided new insights into the ways in which different microbial pathogens incorporate NF-κB modulation as a part of intracellular survival strategies.

## Basic Insights Into NF-κB Regulation From Live Cell Imaging

At the core of the NF-κB pathway are the Rel family of transcription factors: p65 (RelA), RelB, c-Rel, p100/p50, and p105/p52, each containing a central DNA binding motif, known as the Rel homology domain (24). These proteins can form homo- or heterodimers in virtually any combination with p65:p50 dimers appearing to be the most common. In the absence of stimulus, NF-κB activity is suppressed by inhibitor kappaB (IκB) proteins, which anchor NF-κB transcription factors in the cytoplasm. The canonical wing of the NF-κB pathway, defined by the activity of p65-containing dimers, can be activated by diverse stimuli. These range from the proinflammatory cytokines, tumor necrosis factor alpha (TNFα) and interleukin-1 beta (IL-1β), to microbe-associated molecular patterns (MAMPs) like lipopolysaccharide (LPS) and flagellin, which are recognized by surface or phagosomal pattern recognition receptors (PRRs), including the toll-like receptors (TLRs) (25). In each case, activation proceeds via the IκB kinase (IKK) complex, a convergence point for the NF-κB pathway. The IKK complex phosphorylates both NF-κB and IκB proteins (26, 27), regulating the activity of the former and stimulating the degradation of the latter. In the case of IκBα, an IκB isoform associated with the regulation of canonical NF-κB signaling, the protein is phosphorylated at serine 32 and 36, creating a phospho-degron, which is recognized by the E3 ubiquitin ligase complex, SCF^β−*TRCP*^, and leads to polyubiquitination and proteasomal degradation of IκBα (28). IKK-dependent phosphorylation also promotes the degradation of other IκB isoforms (i.e., IκBβ and ε) and the processing of p100 and p105 to p52, and p50, respectively [reviewed in 29).

**Figure 1:**
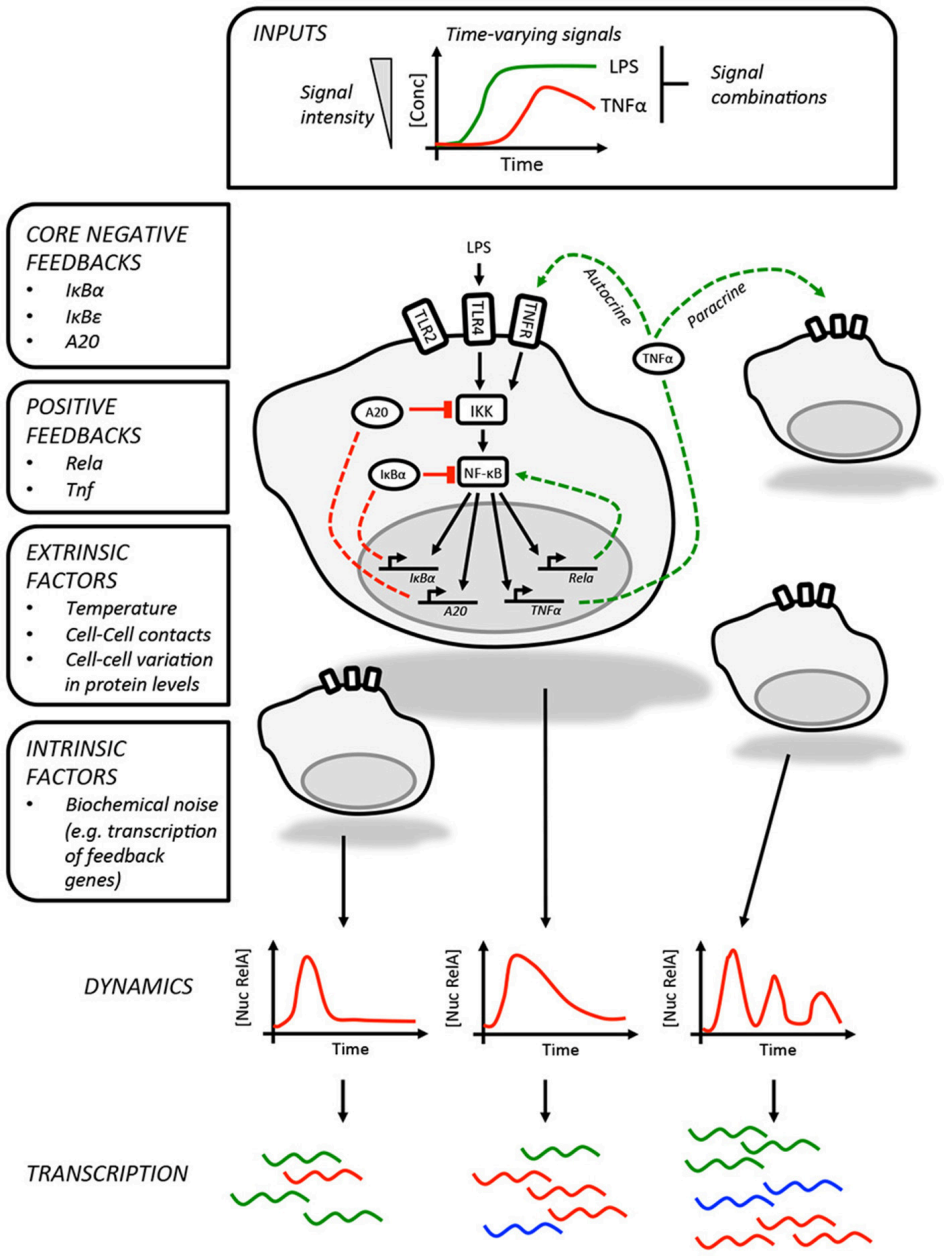
Information processing by the NF-κB pathway. The NF-κB pathway is able to encode information about time-varying stimuli. In this illustration, which depicts LPS-induced NF-κB activity in macrophages, we list the factors that influence the dynamics of the response in individual cells. These include the core negative feedbacks (red dashed lines) and positive feedbacks (green dashed lines). The variability in single cell NF-κB dynamics are contributed to by a variety of factors, including paracrine signaling, and result in different patterns of gene expression between cells. The intrinsic biochemical noise of gene expression will also create variability within the responses of individual cells.

In addition to regulating genes involved in innate immunity and inflammation, p65 also promotes the expression of a core set of negative regulators, IκBα, IκBε, and tumor necrosis factor alpha-induced protein 3 (TNFAIP3/A20, Figure 1) (6, 7, 19). The inherent delay in the expression of these proteins is thought to be responsible for the oscillatory behavior of the pathway. While each of these feedbacks was first identified in genetic and biochemical studies (19, 30–33), the individual roles played by these in shaping NF-κB dynamics was clarified by subsequent studies using live imaging and mathematical modeling. As RNA polymerase II associates with the IκBα promoter prior to stimulation (6), this feedback is rapidly activated on nuclear translocation of p65 and is perhaps most closely linked to the oscillatory behavior of the pathway (7). Expression of IκBε is delayed relative to IκBα and this may play a role in increasing the heterogeneity of the response between cells in addition to helping terminate NF-κB activation after transient stimulation (6, 19). Finally, A20 provides a non-redundant feedback that operates over longer timescales (34), inhibiting IKK activity by antagonizing upstream regulators (35). Expression of the *TNFAIP3* gene, which encodes A20, is temperature sensitive and may imbue the NF-κB pathway with the ability to adjust the expression of select NF-κB-regulated genes across physiologically relevant temperatures during infection and inflammation (36).

The core negative feedbacks are supplemented by additional cell type and stimulus-specific feedbacks. The best example of this is the feedback dominance switching observed in macrophages exposed to LPS (18), which enables cells to discriminate between high and low LPS concentrations. In response to high concentrations of LPS, p65 is able to transactivate expression of the *Rela* gene, increasing the expression of p65 and overcoming negative feedbacks that would otherwise curtail NF-κB activity. This mechanism is likely specific to macrophages or at least lymphoid cells as it requires expression of Ikaros, a transcription factor involved in lymphoid development (37). The NF-κB-regulated expression of TNFα could also be considered a second positive feedback, acting as an autocrine or paracrine signal to prolonging the NF-κB response to LPS in mouse embryonic fibroblasts as well as increasing the heterogeneity of the response in murine macrophages (12, 38).

The challenging task of assigning meaning to NF-κB dynamics has been addressed by recent studies that supplement live cell imaging with microfluidics and transcriptional profiling to either shape and synchronize NF-κB dynamics across a cell population through periodic forcing (10) or link the dynamics in individual cells to single cell RNAseq transcriptional profiles (12). These studies, together with earlier work (6), collectively show that different dynamic responses can produce distinct patterns of gene expression and changes in cellular function. This appears to be because the transcripts of NF-κB target genes with related functions are expressed with similar kinetics or have similar stabilities. In this way, the expression of cytokines and cytokine receptors closely track NF-κB dynamics and will even oscillate, whereas the transcripts for target genes associated with other processes, including remodeling the extracellular matrix, accumulate more slowly and require repeated cycles of NF-κB nuclear accumulation in order to be expressed at biologically meaningful concentrations (10). Therefore, it seems logical that exogenous factors that influence NF-κB dynamics could effectively alter their meaning, impacting gene expression, and potentially compromising the response.

A large number of microbial pathogens are known to utilize effectors that directly target components of the NF-κB system and those that replicate or survive within host cells may also indirectly affect NF-κB as a consequence of other pathogen-encoded activities (39, 40). In most studies, these effects are characterized as simply inhibiting or activating NF-κB signaling in host cells. Given our current understanding of the relationship between NF-κB dynamics and gene expression, we assert that a more nuanced view of these effects is called for if we are to fully understand the role NF-κB signaling plays in innate immunity and host: pathogen interactions.

## Live Imaging as a Tool to Study NF-κB Modulation by Intracellular Pathogens

Overall, the use of live cell imaging to investigate NF-κB responses in cells exposed to live pathogens is surprisingly uncommon and is dwarfed by a wealth of similar studies using purified microbial ligands. Perhaps for this reason, the earliest publications in this area compared the kinetics of the TLR4-NF-κB response in cells co-cultured with intact, extracellular *E. coli* or LPS isolated from the same organism, showing similar effects (41). Other early publications used live cell imaging to correlate the attachment of bacteria to the surface of host cells with the timing of an NF-κB nuclear accumulation and disentangle the asynchronous responses between cells. This was used in two separate studies by the Meyer group to show that *H. pylori* with an intact type IV secretion system could induce p65 oscillations in human gastric epithelial cells (42), and that the force of type IV pilus retraction could stimulate waves of p65 nuclear translocation as *Neisseria gonorrhoeae* microcolonys form and fuse on the surface of infected cells (21).

While these studies using extracellular pathogens have been informative, they are mainly descriptive and do not provide deeper insights into how NF-κB signaling alters during the course of an infection or how it impacts outcome. During intracellular infections, NF-κB activity is very much a double-edged sword that can benefit both host and pathogen. It can strengthen the innate immune response of the host through expression of pro-inflammatory cytokines and directly enhance the microbicidal activity of macrophages by promoting expression of *Nos2* and other markers of M1 polarization (43–45). However, by positively regulating the expression of anti-apoptotic proteins, prolonged NF-κB activation can extend the survival of infected cells, providing a niche for the intracellular persistence and replication of the pathogen. Perhaps for these reasons, a wide variety of bacterial and eukaryotic pathogens, including *Salmonella* (23, 46, 47), *Legionella pneumophilia* (20, 48), and *Toxoplasma gondii* (49, 50) target NF-κB during infection. It is also common for individual pathogens to express multiple effectors, regulating different components of the NF-κB system to contrasting effect, deploying them individually or in combinations at different stages of an infection (39).

**Figure 2:**
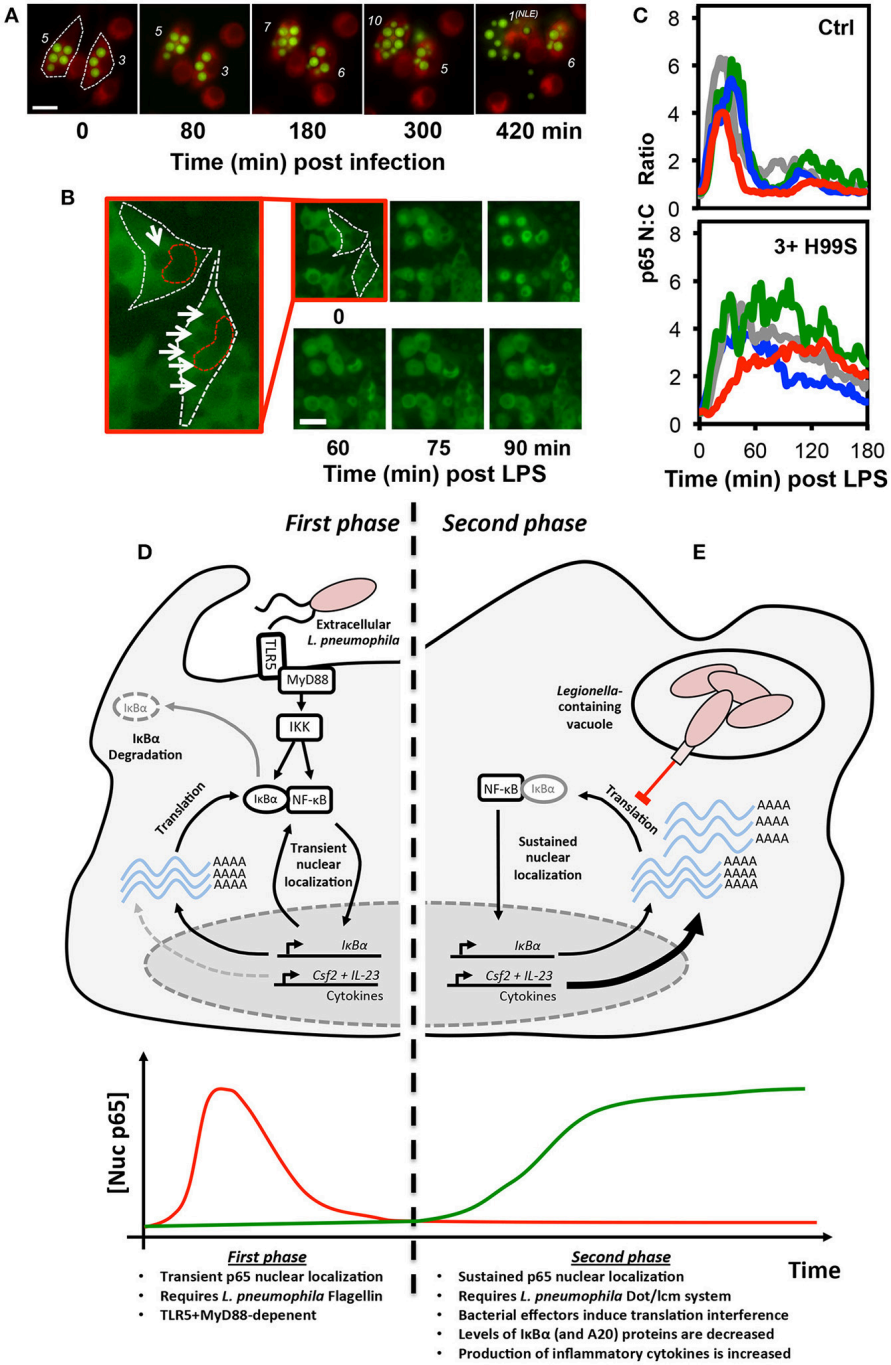
Translational interference by intracellular pathogens alters NF-κB signaling dynamics. Both the fungal pathogen, *C. neoformans* **(A–C)**, and bacterial pathogen *L. pneumophila* **(D–E)**, alter NF-κB signaling by inducing translational interference in host cells. In *C. neoformans* infected cells, these effects are influenced by microbial burden. **(A)** Changes in burden can be tracked in live host macrophages. RAW 264.7 murine macrophages were stained with the membrane dye CellTracker™ Red CMTPX dye (Red) and infected with GFP-expressing *C. neoformans* (Green) then imaged by live cell fluorescence microscopy. The number of intracellular *C. neoformans* in each cell is marked in white. Burden can increase or decrease due to *C. neoformans* replication and non-lytic extrusion (NLE), respectively. **(B)** RAW264.7 cells expressing p65-EGFP were infected with *C. neoformans* and imaged by live cell fluorescence microscopy in the presence of LPS. For the two infected cells, white and red dashed lines indicate cell and nuclear boundary, respectively. Intracellular *C. neoformans* are marked with arrows. **(C)** Quantification of p65-EGFP nuc:cyto ratio in 4 representative non-infected and infected cells (containing ≥3 yeast per cell). Scale bars represents 20 μm. **(D,E)** Epithelial cells exhibit a biphasic NF-κB response to *L. pneumophila*. **(D)** During the first phase, flagellin from extracellular *L. pneumophila* stimulates transient TLR5:MyD88-dependent nuclear localization of p65. **(E)** In contrast, the second phase is flagellin, TLR5, and MyD88-independent and requires the *L. pneumophila* Dot/lcm secretion system. Delivery of effectors into host cells induces translational interference, the partial inhibition of new protein synthesis. This results in a net decrease in the levels of IκBα (and A20) proteins, labile negative regulators of NF-κB signaling. The resulting stable accumulation of p65 proteins in the nucleus promotes increased expression of a subset of pro-inflammatory cytokines, including GM-CSF and IL-23, encoded by the *Csf2* and *Il23a* genes, respectively. The images and data depicted in **(B,C)** were originally published in Hayes et al. (22), reproduced with permission. © The American Society for Biochemistry and Molecular Biology.

Delineating the various events that impact NF-κB activity during intracellular infection can be especially challenging. Intracellular pathogenesis is a multistage process, involving the microbe-active or -passive entry into host cells, intracellular survival of the pathogen, which may be accompanied by replication, and eventual exit (51). Changes in NF-κB activity may be associated with any phase of the process, driven by recognition of microbial antigens by host cell PRRs, either pre- or post-entry, or through the delivery of microbial effectors into the host cell. Even in cell culture models of infection, these events will happen asynchronously and, indeed, intracellular microbial burden will vary between cells. Furthermore, non-infected cells may exhibit so-called bystander effects, either through interaction with shed MAMPs, paracrine signaling, or a combination of both, complicating analysis (23). However, as many intracellular pathogens can be genetically modified to express fluorescent markers or are large enough to be identified in brightfield images, live cell microscopy can be used to track the progress of infection in individual cells while simultaneously monitoring changes in the localization of NF-κB proteins (Figures 2A,B) (22, 23). Quantitative time-resolved measurements of this type, and the ability to separate the responses of bystanders from those occurring in infected cells would be impractical (if not impossible) to achieve using bulk cell analysis techniques.

This approach was used in a recent study by Ramos-Marquès et al. to characterize the effect of *Salmonella enterica* serovar Typhimurium (*S*. Typhimurium) on NF-κB signaling in fibroblasts (23). *S*. Typhimurium is a cause of inflammatory enteric disorders in mammals and is able to colonize fibroblasts after penetrating gut epithelium (52). While it was known that exposure to *S*. Typhimurium was capable of triggering NF-κB activity in these cells through recognition of shed MAMPs, LPS and flagellin by TLR4 and TLR5, respectively, it was previously unclear whether intracellular persistence of the bacterium affected the response. In order to explore this, the investigators used live imaging together with microfluidics in order to transiently expose fibroblasts to live bacteria for 10 min. This approach both limited the exposure of non-infected cells to shed extracellular MAMPs while also minimizing the effects of paracrine signaling. Although infected cells exhibited a heightened initial NF-κB response to *S*. Typhimurium exposure, presumably due to engagement of both surface and intracellular TLRs, subsequent exposure to bacteria or TNFα elicited a much-diminished response. The decreased nuclear translocation of p65 in these cells was accompanied by decreased *IL1B* and increased *SOCS3* expression, a cytokine signaling suppressor. These effects required a functional type III secretion system expressed from the *Salmonella* pathogenicity island 1 (T1) but not pathogenicity island 2 (T2). While the specific *S*. Typhimurium effectors responsible were not identified, it is known that a variety of T1 and T2 effectors directly target NF-κB pathway components and are capable of both increasing and decreasing NF-κB activity in different cellular contexts (47, 53, 54). These include AvrA, which inhibits p65 nuclear translocation by deubiquitinating IκBα (47). The ability to selectively employ combinations of these effectors in different host cell types may provide *S*. Typhimurium with the capability to tune host NF-κB responses to contrasting effect, either leading to the apoptosis of the host cell or extending its viability for use as a growth niche.

## Translational Interference: A Receptor-Independent Mechanism of Altering NF-κB Signaling in Host Cells

Facultative intracellular pathogens, by definition, do not require a mammalian host for replication. It is thought, therefore, that many of the strategies employed by these pathogens to evade host macrophages evolved in order to survive interactions with environmental protozoa, such as amoeba (55). These strategies may involve the expression of virulence factors that enable pathogens to either avoid ingestion by phagocytes or by targeting highly conserved, essential eukaryotic processes within the host cell in order to survive ingestion. It is notable then that a variety of bacterial and eukaryotic intracellular pathogens are able to induce translational interference, the partial suppression of nascent protein synthesis in host cells [reviewed in 56). While the primary purpose of this might be simply to increase the availability of free amino acids within the intracellular environment for microbial growth and attenuate innate immune function, its effects on cellular signaling should not necessarily be dismissed as “collateral” or a secondary effect. As feedback in the NF-κB system requires protein synthesis, translational interference will alter NF-κB dynamics and downstream gene expression. This has been illustrated by experiments where partial inhibition of ribosome function in the absence of external stimulus or microbial pathogens have driven a rapid reduction in IκBα (and slower loss of IκBβ and IκBε) and nuclear accumulation of p65 in murine fibroblasts (57). Within the context of an intracellular infection, this could hypothetically aid the pathogen by disrupting the normal operation of the pathway but it may also provide a receptor-independent mechanism by which intracellular microbial activity could be detected and responded to by host cells. These possibilities have been explored in a number of recent studies utilizing live cell imaging to measure NF-κB activity during infection with the facultative intracellular pathogens, *L. pneumophilia* and *Cryptococcus neoformans* (20, 22).

The encapsulated fungal pathogen, *C. neoformans*, is ubiquitous in urban environments and infects most individuals during childhood. It rarely causes disease in immune-competent hosts (58). Instead, it can enter a chronic, dormant state in host macrophages, often for many years, before later emerging should the immune system become compromised, leading to pneumonia and meningitis. As such, it is generally characterized as an AIDS-associated infection and is thought to be responsible for approximately 181,000 deaths per year worldwide, with most occurring in sub-Saharan Africa where HIV is endemic (59).

The *C. neoformans* polysaccharide capsule is essential for virulence and is largely made up of glucuronoxylomannan (GXM). GXM is synthesized and deployed as capsule rapidly after the inhalation of *C. neoformans* spores, increasing the effective radius of the yeast particle, impeding ingestion by host phagocytes and masking cell wall antigens that could be detected by PRRs. GXM is continually shed during growth as polysaccharide-filled vesicles both pre- and post-phagocytosis and appears to have immunomodulatory activities in this form (60). While there is disagreement in the literature about the precise effects of free GXM (61), possibly due to differences in the cell models used and GXM purification methods, several groups have shown that it is capable of suppressing TLR4 and MyD88-dependent NF-κB activation in a FcγRIIb and SHIP-dependent manner both *in vitro* and in a murine model of endotoxic shock (22, 62, 63).

Interestingly, the effects of GXM and capsular polysaccharides on NF-κB signaling may differ when secreted by phagosomal *C. neoformans*. This was explored in a recent study by Hayes et al. (22), which utilized the RAW264.7 NF-κB reporter cell line first described by Sung et al. (18), in order to simultaneously monitor p65 localization, the expression of an mCherry reporter of *TNF* promoter transactivation, and intracellular microbial burden. During these experiments, microbial burden was highly variable, as *C. neoformans* is able to both replicate within the acidified environment of the phagolysosome and also exit host cells without inducing cell death by non-lytic extrusion (Figure 2A) (64). While phagocytosis of encapsulated *C. neoformans* alone did not have an immediate effect on NF-κB signaling in host macrophages, it was capable of influencing the response of infected cells to pro-inflammatory stimulus. Specifically, when infected cells were challenged with LPS, the amplitude and duration of the response was increased and this was found to be dose-dependent, escalating with intracellular microbial burden (Figures 2B,C). This effect was lost when macrophages were infected with the capsule-deficient, GXM-negative *C. neoformans* mutant strain, CAP59, indicating that this effect was GXM-dependent. Interestingly, only live GXM-positive *C. neoformans* strains but not CAP59 or heat killed yeast induced a measurable decrease in nascent protein production in host cells, as measured by ribopuromycylation, suggesting that the altered NF-κB response was a product of GXM-induced translational interference. These data were consistent with the findings of an earlier independent study showing a reduction in protein translation rate in *C. neoformans*-infected J774.1 murine macrophage-like cells (65). Even though the overall change in NF-κB dynamics in the live cell imaging study were slight and would be difficult to detect in biochemical assays, it seems likely that it would be sufficient to influence the pattern of NF-κB regulated gene expression given the strong association between NF-κB dynamics and transcriptional output, which has been clearly demonstrated in macrophages (12).

The strategies employed by intracellular pathogens to subvert signaling may differ by cell type and can also alter as an infection progresses. For example, *L. pneumophila*, the causative agent of Legionnaires' disease can directly activate IκBα degradation and NF-κB in host macrophages through secretion of LegK1 effector proteins, an IKK mimic (48), promoting host cell survival. However, in epithelial cells, *L. pneumophila* induces biphasic NF-κB activation, which was resolved in a live cell imaging study by the Meyer group (20). The first phase of activation involves the recognition of flagellin, a component of *L. pneumophila* flagella, by TLR5, triggering transient MyD88-dependent nuclear translocation of p65 in infected cells (Figure 2D). This was associated with NF-κB-dependent expression of IL-8, likely benefiting the host (66, 67). The second phase was TLR5 and MyD88-independent and instead required a functional Dot/lcm type IV secretion system, used by the bacterium to deliver effector proteins into host cells from the *Legionella*-containing vacuole (Figure 2E). This stimulated long-lasting, non-oscillatory p65 nuclear localization and was associated with a reduction in IκBα levels and expression of the anti-apoptotic proteins cIAP1, cFLIP, and XIAP, which the authors hypothesized would aid the pathogenesis of *L. pneumophila* through preservation of the intracellular growth niche. It is notable that earlier studies interpreted the TLR5-dependent and Dot/lcm-dependent responses as separate effects achieved at different multiplicities of infection rather than sequential events occurring during infection (68, 69). In this regard, the use of live cell imaging was instrumental in correcting this misconception.

Subsequent studies by an independent group indicate that the second phase of NF-κB activation in *L. pneumophila* infected cells is a product of translation interference (40), requiring the *L. pneumophila* Dot/Icm type IV secretion system to deliver a cocktail of five bacterial effectors into host cells to globally decrease—but not completely inhibit—mRNA translation. As IκBα proteins are particularly labile and turn over quickly, under these conditions, the rate of IκBα degradation exceed the rate of production, resulting in a rapid decrease in IκBα protein levels accompanied by stable nuclear accumulation of NF-κB in host cells. This results in the selective “superinduction” of specific transcripts that are not normally responsive to transient PRR-mediated NF-κB activity. While the precise mechanism remains unclear it seems likely that the shear number of these transcripts and possibly the stability of the protein products overcome the translational bottleneck in *L. pneumophila* infected cells. Proteins upregulated in these cells included the proinflammatory cytokines, interleukin-23 and GM-CSF, suggesting that this stable nuclear localization of p65 may not be entirely beneficial to the pathogen and may represent a receptor-independent mechanism of NF-κB activation, providing a means to initiate an innate immune response.

## Concluding Remarks

Live cell imaging has transformed our understanding of how the NF-κB system coordinates the cellular response to stimuli, especially in innate immune cells. The ability of this technique to disentangle differing and asynchronous responses of individual cells has also made it ideal for investigating how intracellular pathogens manipulate NF-κB signaling in host cells, particularly in instances where the effects on this pathway are influenced by intracellular microbial burden or the changing repertoire of microbial ligands and effectors presented or deployed during the course of an infection (22). Despite the various advantages of the technique, to the author's knowledge, it has seldom been used for this purpose and this mini-review represents a relatively complete overview of the current literature in this area.

Prior genetic and biochemical studies have shown that modulation of host cell NF-κB signaling is relatively common among gastrointestinal pathogens, including *Helicobactor pylori* (70), *Shigella* (71), and *Yesinia* (72), and has been demonstrated in other invasive bacteria, such as *Mycobacterium tuberculosis* (73). Overall, it appears that NF-κB modulation is utilized by pathogens to either “buy-time” for intracellular replication, as employed by *Mycobacterium tuberculosis* and *Shigella* (71, 73) by stimulating the expression of pro-survival NF-κB-responsive genes, or to do quite the reverse, by using effectors that inhibit host cell NF-κB-activity to blunt an inflammatory response or promote apoptosis in order to evade destruction by innate immune cells or aid escape and dissemination (72). While these previous studies have successfully identified the molecular players required for subversion of NF-κB signaling in host cells, a reexamination of these effects using live cell imaging is merited. As demonstrated by the research highlighted in this review, this method could help to resolve otherwise hidden bi- or multiphasic responses to intracellular pathogens (20), and perhaps most interestingly, link the different NF-κB responses of individual cells to specific transcriptional responses using fluorescent reporters (18, 22) or downstream single cell transcriptomics (12) and different infection outcomes (e.g., intracellular replication, non-lytic exocytosis, host cell death, killing of the pathogen etc.).

Despite the potentially very useful insights that can be obtained through the application of this technique, our enthusiasm should be tempered by an awareness of its inherent limitations, which stem from the absolute requirement to modify the system being studied through the use of fluorescent tags and the over-expression of exogenous proteins, both of which have the potential to affect the behavior of the pathway. The former is perhaps least concerning as careful characterization of p65 fluorescent fusions has suggested that GFP-tags neither interfere with the ability of the protein to transactivate gene transcription or correctly associate with regulators, including the IκB proteins (74), although it may have as yet unrecognized consequences. The effects of protein overexpression on the behavior of the NF-κB are less clear-cut. Experimental evidence has suggested that p65 overexpression has little effect on pathway behavior (75), although separate studies have indicated IκBα levels recover more rapidly after stimulation in cells expressing p65-GFP in addition to the endogenous protein (42). More recent studies have attempted to minimize the effects of overexpression by using BACS or stable transduction of viral constructs to express p65 fusions under the control of the endogenous promoter (18, 36). In these ways, expression levels of tagged p65 can be regulated appropriately by the cell and kept more closely to endogenous levels than might be achieved through transient transfection of plasmid constructs. It also seems likely that future studies will utilize CRISPR/Cas9-based gene editing to introduce fluorescent proteins into the endogenous locus of NF-κB genes to avoid protein overexpression.

## Author Contributions

The manuscript was designed by DN and written by DN with assistance from RN. All authors read and approved the final manuscript.

### Conflict of Interest Statement

The authors declare that the research was conducted in the absence of any commercial or financial relationships that could be construed as a potential conflict of interest.
